# Perception of Pediatric Nurses on the Use of Standardized Nursing Handover Process in Intra-Hospital Patients Transfer: Attitudes, Barriers, and Practical Challenges

**DOI:** 10.3390/nursrep14040272

**Published:** 2024-11-28

**Authors:** Irene Martínez-Muñoz, José Luis Díaz-Agea, Jesús David Pastor-Rodríguez

**Affiliations:** 1 Health Sciences PhD Program, Universidad de Murcia, Campus de Espinardo of Murcia, 30100 Murcia, Spain; irene.martinez7@um.es; 2 Pediatric Intensive Care Unit, Hospital Clínico Virgen de la Arrixaca, 30120 Murcia, Spain; 3 Facultad de Enfermería, Universidad de Murcia, 30120 Murcia, Spain; jesusdavid.pastor@um.es

**Keywords:** patient handoff, patient transfer, qualitative research, intensive care, hospitalization area pediatrics

## Abstract

Standardized transfer is an evidence-based framework designed to improve communication between healthcare professionals, reducing risks and ensuring safe, high-quality care. Despite its benefits, implementing this framework in clinical practice poses challenges. Nurses often do not use a systematic guide as a theoretical framework for handovers in daily practice. Objective: To explore nurses’ perceptions regarding the use of standardized transfers. Methodology: This exploratory qualitative cross-sectional study aimed to gain insight into nurses’ experiences and perspectives on pediatric patient transfers. Using purposive sampling, nurses from the pediatric intensive care unit and hospital wards at the hospital institution hosting the study were interviewed. Data were collected through 21 in-depth individual interviews conducted between April and May 2023. The semi-structured interviews, lasting 16 to 28 min, focused on nurses’ views on communication between units during patient transfers. The qualitative approach allowed for a comprehensive understanding of nurses’ perceptions, particularly the barriers they face in practice. The study included 21 nurses: 9 from the pediatric intensive care unit and 12 from pediatric wards. To ensure diverse representation, nurses with varying levels of work experience were included, and at least one nurse from each hospital ward participated. Results: The data were classified into the following main categories: the current state of pediatric patient transfers, attitudes of healthcare professionals, barriers and challenges to implementation, nursing documentation, motivational aspects, and the child-family relationship. The findings revealed significant issues in the communication process during patient transfers, with no systematic guidelines in place. While nurses demonstrated a positive attitude toward the standardization of transfers, they identified numerous practical challenges, particularly those related to the hospital’s nursing documentation system. Conclusions: Nurses view standardized transfers favorably, but they face substantial barriers that limit their practical implementation.

## 1. Introduction

In hospital care, the transfer of patients between care units is one of the most routine yet critical practices. This procedure demands careful planning and preparation by the nursing team involved, following a standardized protocol to minimize risks and ensure the continuity of high-quality care [1,2]. Transfer is defined as the verbal and/or written communication of relevant information between the nursing staff of the discharging unit and the receiving unit [3]. Handover is a critical communication process involving the transfer of essential patient information and the responsibility for patient care from one provider to another. This process includes not only the exchange of information but also active interaction between the sender and receiver to clarify concepts, align on procedures, and confirm strategies that support the continuity and safety of nursing care. Effective handover requires organized strategies to ensure the accurate exchange and verification of information, minimizing potential risks and promoting seamless patient care [4]. Nurses, as primary caregivers, play a central role in this process, assuming responsibility for ensuring patient safety and care continuity during handovers [5].

An inadequate or incomplete transfer introduces risks, compromises patient safety, and increases the likelihood of nursing errors due to care interruptions [6]. This is particularly significant when the transfer involves patients moving from the Intensive Care Unit (ICU) to other hospital units (HCU), as these transfers are more complex. In addition to the transfer itself, there is often a de-escalation in the intensity of care and monitoring, which requires even more meticulous communication [7]. Additional challenges include the nature of the information, which tends to be more technical and specialized. In this context, the discharge of complex patients is considered a critical moment for patient safety [8]. For these reasons, standardized transfers have been identified as a key indicator in care quality standards aimed at optimizing the safety of patients transitioning from the ICU to other hospital units [9,10].

Ensuring quality information exchange between the ICU and other hospital areas is essential for maintaining continuity of care. This fosters comprehensive nursing care and enhances both patient and family satisfaction, as well as nurses’ perception of the care they provide. Consequently, handovers have become a central focus in national health strategies for improving patient safety. A review of the literature reveals that handovers in clinical practice continue to pose a significant threat to patient safety. Communication failures account for 70% of adverse events in healthcare, with 50% of these failures occurring during the handover process [1,2,5]. There are several obstacles that contribute to ineffective communication. First, handovers often occur in environments that do not meet the basic requirements necessary for effective communication. In addition, the individual nature of information exchange can further complicate the process. These factors can lead to poor communication between nurses, resulting in errors in the content of the information and incomplete or inaccurate data about the patient’s condition or care, ultimately putting patient safety at risk. Improper handovers can lead to numerous adverse outcomes, including incorrect treatment, medication errors, prolonged hospital stays, and discrepancies in patient data [11,12].

There is a commonly perceived negative attitude toward patient transfers, which may stem from the various challenges associated with this nursing process. Studies have identified barriers such as the complexity and adequacy of the information being shared, differing family expectations regarding care at various levels, the time required for preparation, and unclear or overly detailed communication formats. These limitations contribute to frustration and negative attitudes toward the transfer process [13].

A review by Vázquez Calatayud et al. underscored the concerns nurses face during patient transfers, particularly highlighting feelings of anxiety and stress, which were exacerbated among less experienced nurses [14]. In a qualitative study by Stephen James et al., nurses expressed concerns regarding the timing of transfers, the content and method of communication, and interactions with family members. The study emphasized that for nurses, patient transfer is a crucial moment that requires careful planning to ensure the continuity of care for complex patients. Challenges cited included the communication format, timing, and overall organization of the transfer process [15].

Similarly, Ronny Enger et al. explored nurses’ perceptions during patient transfers and concluded that transfers from the ICU are perceived as significant challenges due to inadequate planning and poor reporting procedures. Their study highlighted the need for improvements, such as allocating time for proper reception planning and implementing protocolized, concise, and clear reporting [16].

The findings of Madeleine Powell further reinforce these concepts. Her study highlights that critical clinical information was sometimes inaccurate or omitted, which is a significant issue as such communication gaps compromise the continuity of care and patient safety. The literature emphasizes the challenges associated with patient handovers. Nurses have identified several barriers to effective handover, including interruptions, lack of planning, heavy workloads, and the absence of a standardized handover system [17].

Despite ongoing research, patient handover remains a persistent challenge for nursing staff. One proposed solution is the standardization of the nursing handover process, which could be a pivotal strategy in enhancing patient safety. Standardization improves the clarity and quality of communication, thereby increasing the overall quality of care and minimizing the risk of inconsistencies [17,18,19].

Implementing standardized communication processes is crucial to reducing the risks inherent in unstructured handovers, ultimately fostering a safer environment for patients. Excellence in discharge planning should therefore be a priority to ensure patient safety [20]. Addressing communication-related issues requires leadership and management to actively promote initiatives aimed at standardizing patient transfers. This is essential for delivering high-quality, safe care [21]. Given the critical nature of patient transfers, prioritizing standardization to enhance quality should be a key objective. Healthcare managers must acknowledge the significance of this issue and implement interventions to ensure the safe transition of patients. Effective organization, a safe and supportive work environment, and engaged staff lead to improved patient safety and care quality [22].

However, despite these recognized needs, the ideal of standardized transfer systems has not yet been fully realized in our healthcare system. A standardized, systematic approach to patient transfers, devoid of variability based on individual or contextual factors, would significantly enhance hospital care [23,24,25].

Evidence suggests the existence of various structured and standardized frameworks for patient handover. One such model is the SBAR (Situation, Background, Assessment, and Recommendation) framework, which has evolved over time into other models, such as ISBAR (Identification, Situation, Background, Assessment, and Recommendation) and ISBARQ (which includes a “Q” for Questions). The implementation of these standardized frameworks helps address communication gaps during information transfer, ultimately improving patient care and reducing complications associated with communication failures [26,27,28].

A systematic approach to information transfer is critical for promoting safe, high-quality continuity of care, yet its practical implementation remains a challenge. Healthcare professionals must advocate for the necessary tools to achieve these quality outcomes in practice [29,30]. Studies suggest that numerous barriers still hinder the effective execution of this procedure. It is therefore vital to understand the perspectives of nursing professionals involved in patient transfers to reach consensus on strategies that ensure safe and efficient handovers between units [31].

The objective of this study is to explore the perceptions of nursing professionals regarding the transfer of pediatric patients from the Pediatric Intensive Care Unit (PICU) to other pediatric hospitalization units. Given that qualitative methodology enables an in-depth exploration of participants’ experiences and perspectives (emic perspective), it is particularly well-suited for identifying strengths and weaknesses within the current transfer process. This approach allows nurses to describe their experiences, share opinions, and highlight areas for improvement, with the aim of developing a standardized tool to facilitate patient handover. Understanding the perspectives of staff involved in these transfers is essential for optimizing the process and transforming it into an efficient, routine practice that supports continuity and safety of care. To achieve this, an exploratory qualitative study was designed using individual interviews to gain a deeper understanding of the nursing staff’s perceptions regarding patient transfer procedures.

This study is based on the assumption that nursing communication during the transfer of patients from the PICU to inpatient wards is suboptimal due to the absence of a standardized protocol. Furthermore, the variation in nursing records and the use of different software systems across units hinders the effective transfer of information.

## 2. Materials and Methods

The principal investigator adhered to the COREQ checklist for qualitative research throughout the study [32].

### 2.1. Study Design

To address the primary objective, an exploratory, cross-sectional qualitative study was conducted to gain a deeper understanding of nursing professionals’ experiences and perspectives on pediatric patient transfers in the healthcare setting. Qualitative methodology was selected as it allows for an in-depth exploration of participants’ perspectives (emic perspective) regarding quality transfers between different levels of care [33].

The purpose of this methodology is to understand the realities faced by nurses, as viewed from their own experiences, so that we can explain and address the questions posed in the study. The aim is not to make predictions but to gain a deeper understanding of the phenomenon [34].

Qualitative research provides the necessary tools to study the subject in a holistic context. Rather than dealing with quantitative data or statistical analysis, this study focuses on exploring the perceptions of nurses as they see and experience the transfer process. Our objective is to investigate nurses’ opinions and thoughts on the communication between pediatric ICU staff and hospital wards, with the nurses themselves offering insights into this reality [35,36,37].

To achieve this, open data collection techniques were used, allowing for deeper exploration of key aspects. Unlike quantitative methods such as surveys, which limit responses to predefined questions, qualitative methods provide the flexibility to delve into the complexities of participants’ experiences [37].

### 2.2. Participants

Participants were selected based on the study’s focus. Nurses working in pediatric hospitalization units and the Pediatric Intensive Care Unit (PICU) at the hospital institution hosting the study were chosen for accessibility and relevance to the study topic. Theoretical or purposive sampling was used [36], aiming to capture the widest variability in the sample to reflect a comprehensive range of perspectives.

The predefined sampling criteria included:

- Nurses from different pediatric hospitalization units to represent the needs of all services in the area.
- Nurses with varying levels of experience.
- Efforts were made to ensure representation from all these sampling categories.

Inclusion Criteria:

- Nurses (both pediatric nursing specialists and non-specialists) working in pediatric hospitalization units or the PICU at the time of the study.
- Nurses with more than one year of experience in their field (irrespective of the department or hospital they had previously worked in).
- Voluntary participation.

Exclusion Criteria:

- Failure to sign the informed consent form.
- Nurses with less than two years of experience as nurses.
- Nurses working in areas other than pediatric hospitalization as nurses.

The flexible nature of qualitative research design made it difficult to predetermine the number of participants or whether their characteristics would change as the interviews progressed.

### 2.3. Data Collection

Data collection was performed using individual in-depth interviews. The use of focus groups was rejected because, in general, focus groups require participants who are not familiar with each other or the moderator, a criterion that was not met in this case [33]. Pediatric hospital settings often involve pre-established professional and personal relationships, which could influence responses and distort the discourse.

Additionally, as the topic might generate conflicts among staff, the individual in-depth interview was considered more appropriate. This method fosters a more private and intimate environment, allowing participants to express their thoughts, experiences, and feelings without the influence of colleagues [33,34,36].

Given the aim of understanding nurses’ perceptions, personal interviews were considered the most suitable tool to capture their experiences regarding pediatric patient transfers. The holistic nature and flexible structure of the interviews enabled participants to describe their reality in their own words.

Prior to fieldwork, the necessary permissions were obtained from the healthcare institution, along with confidentiality agreements from the participants. Fieldwork took place between April and May 2023. The interviews were conducted by the principal investigator of this study. Participants were selected through purposive sampling, allowing the researchers to target nurses with specific characteristics relevant to pediatric patient transfers. The sample included nurses from both the PICU and pediatric hospitalization units at the hospital institution hosting the study, chosen due to their direct involvement in the patient transfer process.

Selection criteria ensured that the sample captured a diverse range of experiences and perspectives. Nurses were recruited from different hospitalization units, and the study included participants with varying levels of experience, enabling a comprehensive understanding of both seasoned professionals’ insights and those of newer staff members.

Participants were recruited through direct personal contact. The study’s objectives, the confidentiality of the data, privacy aspects, and the voluntary nature of participation were explained, emphasizing the importance of their collaboration. No participant declined the interview after being informed about the study. Before each interview, participants signed an informed consent form, and their permission was obtained to record the interviews for transcription and analysis.

Interviews were scheduled at mutually agreed times and conducted in a designated hospital room for practical reasons. The interview duration ranged from 16 to 28 min, depending on the depth of the topics discussed. This approach ensured the participation of nurses well-positioned to provide in-depth insights into the challenges and experiences surrounding pediatric patient transfers.

In total, 21 semi-structured interviews were conducted. As Vallés [36] notes, these interviews consist of a series of questions structured by the researcher according to the study’s objectives but delivered in a free and non-directive manner. The interviews were guided by a script (Table 1). Open-ended questions were asked, and any subtopics not mentioned spontaneously by the participants were addressed by the interviewer.

During the interview process, where only the researcher and the participant were present, efforts were made to create an atmosphere of intimacy and familiarity, similar to a natural conversation. This approach aimed to build sufficient trust between the participant and the interviewer, encouraging the participant to share their experiences in an open and authentic manner, externalizing their impressions. Achieving this climate of rapport was challenging, as it requires experience [33,34,36].

In-depth interviews were conducted until no new significant information emerged that would contribute to a deeper understanding of the study topic. However, due to the researcher’s limited experience, it cannot be conclusively stated that theoretical saturation was fully achieved. After conducting 21 interviews, no additional data were provided that introduced new perspectives on the topic.

### 2.4. Data Analysis

All interviews were audio-recorded and transcribed verbatim for a comprehensive analysis. The data were analyzed using discourse analysis, which allows for the identification of patterns and themes within spoken text. In line with an inductive approach, the transcripts were initially reviewed multiple times to develop a deep understanding of the content. The first step of the analysis involved fragmenting the data and assigning preliminary codes, which were created based on repeated words, phrases, or concepts that appeared significant to the participants’ experiences.

Once initial codes were generated, two researchers independently applied these codes to the transcripts. This coding stage involved categorizing specific segments of text that illustrated similar ideas into broader themes. For example, repeated comments related to communication barriers or positive feedback on standardized procedures helped form overarching categories. These preliminary themes were refined through iterative discussions between the researchers, who compared and synthesized their findings to ensure consistency and cohesion in the analysis. This process facilitated the identification of five main themes that aligned with the study’s objectives, namely: the current state of pediatric patient transfers; attitudes of healthcare professionals; barriers and challenges in implementation; nursing documentation; and motivational aspects.

The two researchers independently analyzed and coded the data, ensuring inter-coder reliability by regularly discussing discrepancies. The collaborative process led to the integration of shared perspectives, which further validated the thematic structure. After achieving a consensus on the coding scheme, the final categories were defined. This process ensured that the data were rigorously analyzed and represented an authentic and accurate reflection of participants’ experiences.

The final themes were reviewed to verify alignment with the study’s focus on pediatric patient transfers, particularly regarding motivational aspects and standardized handover benefits. This thematic approach provided a structured lens to capture and present the rich insights shared by the nurses, while also allowing the primary researcher to carefully remain aware of any preconceived notions during the coding and categorization stages

### 2.5. Quality Criteria

The quality of the study was assessed according to the criteria outlined by Calderón [38], ensuring methodological rigor across four key areas:

Epistemological adequacy: the qualitative approach was selected as the most appropriate method to gain insight into the reality of pediatric patient transfers as perceived by nurses. This approach allowed for a comprehensive, contextually grounded exploration of the nurses’ firsthand experiences, enabling a deeper understanding of complex interpersonal and procedural dynamics that would not be captured through quantitative methods.

Relevance: understanding nurses’ perceptions of communication during pediatric transfers is crucial for identifying areas of improvement and integrating standardized tools into daily pediatric care practice. To ensure relevance, the study focused on aspects directly related to clinical communication, continuity of care, and patient safety, areas essential to high-quality pediatric care.

Validity: data validity was ensured by designing the interview guide to address key study objectives while allowing participants to share their insights openly. In-depth interviews provided rich, nuanced data on the nurses’ perceptions, and analysis was conducted with careful attention to identifying genuine patterns and themes, preserving the authenticity of the participants’ perspectives.

Reflexivity: the interviewer maintained an open and flexible stance, actively working to minimize personal biases and preconceptions. Reflexivity was practiced throughout data collection and analysis by continuously reflecting on and acknowledging any personal influences that could impact interpretation. Regular peer debriefing sessions also supported a balanced analysis, ensuring that the findings accurately reflected the participants’ experiences.

### 2.6. Ethical Considerations

All relevant ethical standards were followed in conducting this study. The study was reviewed and approved by the Research Ethics Committee of the hospital institution hosting the study, which issued a positive resolution on 27 April 2023 (Verification code: CARM-4e81e188-e-4ed-0179-1dcc-0050569b6280).

Each nurse participating in the study was fully informed about the research objectives, and signed informed consent was obtained voluntarily after ensuring that participants understood the provided information. The anonymity of all data was maintained in compliance with Organic Law 3/2018, dated 5 December, on the protection of personal data and the guarantee of digital rights.

The study adhered to the standards set forth in the Declaration of Helsinki for clinical research, ensuring full respect for the fundamental rights of all participating nurses.

## 3. Results

The results obtained are presented below:

As outlined in the methodology section, 21 in-depth interviews were conducted, ensuring representation from nurses working in both the Pediatric Intensive Care Unit (PICU) and various pediatric inpatient units. At least one nurse from each unit participated; nine nurses were from the PICU, and twelve were from the inpatient units. The sample also encompassed nurses with varying levels of experience. The perceptions of the nurses regarding the pediatric patient transfer process in healthcare practice are presented below, categorized according to the framework outlined in the methodology section.

The data was classified according to the following main categories:

- Pediatric handover, where do we stand?;
- Attitude of professionals;
- Barriers and difficulties in implementing its use;
- Own nursing records;
- Motivating aspects

### 3.1. Pediatric Handover, Where Do We Stand?

The transfer of pediatric patients remains a significant challenge in healthcare. This study provided an opportunity to capture the perceptions of the nursing teams across different pediatric care areas.

The current patient handover process is not standardized, leading to significant variability depending on the individual nurse’s discretion, who, influenced by their context, decides what information to convey.

“*Right now, it is not protocolized; we only receive the information the transferring nurse deems important.*”

(I: 1)

“…*I’ve been left at the end, having had a bit of an argument*…”

(I: 16)

This lack of standardization results in an information handover that many participants describe as inadequate. For example, ICU nurses believe they are sharing relevant information but admit uncertainty about whether it is sufficient or meets the needs of the nurses on the inpatient wards.

“…*We should ask the inpatient staff what information would be most useful and how they would prefer to receive it.*”

(I: 19)

In contrast, hospital nurses feel that essential information is often missing during handover.

“*…It seems to all of us that the information at handover is very limited…*”

(I: 5)

“*…The handover happens in person, but I feel that information is lost along the way…*”

(I: 8)

Nurses emphasized the critical importance of effective communication during patient transfers but acknowledged confusion about what exactly should be conveyed. They called for consensus to eliminate inconsistencies in the communication process.

“…*We don’t follow a structured system like we discuss in theory that would allow for an effective or quality transfer between health professionals. As a result, key information is sometimes missing*…”

(I: 9)

“*I tell you what I feel is important to tell you*…”

(I: 21)

“…*I might not realize the importance of something that could be vital for you on the ward, and I often simplify too much*…”

(I: 12)

Currently, patient transfers are conducted verbally. While a transfer form is available, it is often seen as inadequate and not particularly useful. Nurses perceive it as a task imposed by management rather than a tool to improve care, leading to inconsistent use and the perception of it as an extra burden.

“*The transfer is verbal; I write down notes about the child’s condition and medication on a piece of paper because no one looks at the official transfer form.*”

(I: 16)

“*The transfer form doesn’t motivate us because it’s not useful. It’s just filled out to meet targets.*”

(I: 3)

“…*The transfer form is very basic. It’s inappropriate, with little information about treatments*…”

(I: 18)

Some nurses even admitted to being unaware of the form’s purpose.

“…*I think many people don’t even know that it’s supposed to be filled out, or they don’t actually use it.*”

(I: 8)

There is currently no well-structured planning for the transfer of pediatric patients between units. The nursing staff expressed a need for improvements in this area to enhance patient safety and continuity of care.

“*We need a protocol that ensures everyone, from ICU to ward staff, knows what to do, what to record, and that everything is done consistently.*”

(I: 4)

### 3.2. Attitude of the Professionals

Due to the lack of standardized planning, the transfer process is not always conducted under optimal conditions, which can lead to frustration among the staff. Factors such as workloads and varying levels of experience contribute to this.

“*My feeling is that they’re not really interested. If you go into detail, they don’t care*…”

(I: 1)

“…*Sometimes they don’t even listen. I end up saying, ‘If you have any questions, just call me.*”

(I: 3)

“…*It feels like you’re a nuisance, like you’re nobody, and that’s disheartening when you’re the nurse who knows the patient best.*”

(I: 14)

This lack of systematization has relegated the transfer process, considered vital by the nursing staff, to a secondary role in practice, with priority given to direct patient care rather than communication.

### 3.3. Barriers and Difficulties in Implementation

The nurses interviewed identified several barriers they currently face. These include issues with existing computer records, which do not support the continuity of care during patient transfers, challenges related to the timing of patient transfers, variations in the work experience of the nursing staff, and the lack of a standardized process for information exchange.

The most significant obstacle, as described by all participants, is the nursing documentation system. In pediatric care, different nursing documentation systems coexist, preventing nurses in inpatient units from accessing data recorded by the care team during the child’s stay in the ICU.

“*The problem we have is that our communication systems are different, so I cannot access their data, and they cannot access mine when I send them a child. It’s a significant handicap when there is no proper communication because we don’t even have a shared tool. If you can’t tell me, I can’t access it. Many things are left unclear, leading to errors due to lack of communication.*”

(I: 1)

This lack of access complicates the continuity of care, as nurses are unable to see the care provided or medications administered during the child’s ICU stay. As a result, the receiving nurse relies solely on verbal information during transfer.

“*We didn’t know what they did in the ICU because we can’t access their notes.*”

(I: 3)

“*Maybe they tell you what the patient took that day, but from start to finish, I don’t know*… *They should provide a sort of summary.*”

(I: 14)

“*When the child arrives, you can’t review the full history, so it feels like a blank period.*”

(I: 2)

Although a transfer form is used, it only includes limited information, such as the latest vital signs and devices the child is using. Many other essential aspects of care, which are critical for ensuring safe and effective continuity, are missing from this form, limiting its usefulness in clinical practice.

“*The transfer register doesn’t seem adequate. It feels like it’s completed for the sake of it, but I don’t find it very useful. I would modify it to make it a tool that is actually helpful.*”

(I: 7)

There is a widespread call for a unified system that collects all relevant data in writing and serves as a clear reference during transfer. At the same time, nurses highlight the need for inpatient nurses to access a child’s ICU progress and propose incorporating the nursing handover into a shared computer system, a challenge for management.

“*We need a unified register where we can see everything or collaborate.*”

(I: 4)

“*If we had a standardized, effective handover process with clear steps and critical information, it would help us better understand the patient’s situation.*”

(I: 7)

Another challenge mentioned is the timing of the transfer, which is perceived differently depending on the role. Some nurses suggest that understanding the transfer from the perspective of the receiving nurse would help improve communication.

“*I hadn’t worked much in inpatient care before, so I didn’t pay as much attention to that call as I do now.*”

(I: 9)

ICU nurses occasionally report feeling that they are not being listened to, which diminishes their interest in the transfer process. Additionally, the nurse-to-patient ratio in inpatient areas is higher, making it essential to coordinate transfer times so that the receiving nurse can give full attention to the process.

“*Maybe you bring the child, but it’s not a good time for them. We should try to agree on a suitable time.*”

(I: 9)

“*You try to agree on it, but after waiting for hours, I may not be able to wait any longer because the ICU needs the bed.*”

(I: 12)

Experience level is another factor influencing the quality of information transfer. More experienced nurses are often better at providing detailed information, but standardizing communication could address this variability.

“*It often depends on the person. You can tell when someone has more experience because they miss fewer details.*”

(I: 8)

“*We sometimes assume the other person understands, but we don’t always know their experience level or whether they need more explanation.*”

(I: 14)

“*It’s not the same handing over to a colleague with years of experience versus someone new or with a different background.*”

(I: 1)

It is worth noting that only two interviewees mentioned standardized handover systems. Most nurses, while recognizing the importance of guidelines for quality transfers, were unaware of these systems. This lack of awareness could be due to limited training in this area.

“*I don’t think we’ve had training in patient transfers. Nursing education hasn’t emphasized the importance of this process.*”

(I: 5)

“*We don’t have a foundation where the significance of transfer has been instilled. Maybe with a generational shift, future nurses will come with a different mindset.*”

(I: 7)

Training and the implementation of guidelines are needed to standardize the handover process.

### 3.4. Nursing Records

There is no standardized nursing discharge that outlines the nursing care provided or offers a care guide to follow. Continuity is primarily based on the medical discharge report, which does not include nursing-specific details.

“*You can refer to the medical report, but it’s not the same as a daily nursing progress report, especially since we are the ones at the bedside.*”

(I: 2)

“*There’s no nursing discharge report, only one from the doctor, so we have to make a bit of noise about it.*”

(I: 5)

“*The doctor gives a report, but then you have to write the medications on a piece of paper. Is that nursing?*”

(I: 3)

Nurses are often forced to rely on parents for information about the care their child has received.

“*You discreetly ask the mother when certain treatments were given.*”

(I: 14)

The absence of a shared nursing communication tool across units leads to a feeling of discontinuity and a gap between ICU and ward care. Recording relevant data during transfers would allow for easier access and review in case of uncertainties.

“*If something is missed during handover, you could just check the notes and see what happened in the previous days.*”

(I:13)

### 3.5. Motivational Aspects

Nurses express a positive attitude toward the implementation of standardized handovers, believing it would improve patient care, enhance safety, and empower the nursing profession by promoting care based on its unique body of knowledge.

“*I believe nursing is ready to be part of any project that improves this issue.*”

(I: 1)

“*A refresher is needed. We get caught up in day-to-day tasks, but I think people would be motivated by reminders of these important points.*”

(I: 10)

“*I think everyone wants to work safer*…”

(I: 17)

They also emphasize that management should recognize the existing challenges and support the implementation of standardized systems for safe, high-quality transfers, incorporating both verbal and computerized records of all nursing care.

### 3.6. Benefits of Using Standardized Handover

Nurses unanimously agree that standardizing the handover process would benefit patients, streamline care practices, and strengthen the profession as an autonomous discipline. Accessing previous care information would reduce the risk of errors in medication administration, tests, and treatments. Moreover, it would eliminate the need to rely on parents for information, which nurses consider unprofessional.

“*Standardized protocols would improve care. When you work with a protocol, there are fewer mistakes.*”

(I: 14)

“*It would mean greater quality of care and greater safety*…”

(I: 3)

“*It would help us to know what care has been given to the child and not have to ask the parents. I think it would greatly benefit the quality and safety of child care.*”

(I: 17)

## 4. Discussion

This study aimed to explore the perceptions of nursing staff regarding the transfer of pediatric patients from the Pediatric Intensive Care Unit (PICU) to inpatient units.

Despite existing literature demonstrating that standardized handover protocols enhance the quality and safety of patient care, their implementation as a tool to streamline care practices remains a challenge for management teams. One of the strengths of this study is that it incorporates the perceptions of both pediatric ICU nurses and those from various hospitalization services, enriching the results by providing a broader perspective and allowing for comparisons between different viewpoints.

The data collected indicate that the nurses in the pediatric department at the hospital institution hosting the study recognize significant deficiencies in communication between the different hospitalization units. All participants highlighted this issue, emphasizing the need to improve the transfer of information across levels of care. These findings align with previous research [16,18].

The study reveals a range of barriers to effective handover, which pose genuine obstacles to ensuring continuity of care. A major issue is the absence of a unified electronic record that tracks the most critical aspects of a child’s care and development. Currently, various nursing documentation systems are used at different stages of care, preventing hospital nurses from accessing the full range of care provided in the ICU. This impedes the ability to monitor admitted patients and verify essential care information, increasing the risk of errors and compromising patient safety. Addressing this issue is a pressing challenge, and the nurses unanimously call for a resolution. Furthermore, the available electronic tools are often incomplete and seen as impractical, used more to meet managerial objectives than to facilitate care. As not all nurses are familiar with these tools, their use is not standardized, and they are perceived as an additional, ineffective burden.

Although the nurses interviewed expressed enthusiasm for the implementation of standardized handover procedures, acknowledging the benefits for both the profession and patient care, they identified several challenges that must be addressed to implement such systems effectively. Nurses expressed a strong interest in being actively involved in developing strategies for a standardized transfer system, unanimously recognizing the need for standardization to ensure safe, high-quality continuity of care [39].

Standardizing the handover process would clarify nurses’ professional roles and serve as a method to validate their contributions. The barriers identified in this study—related to technology, workload management, professional experience, and the absence of clear guidelines—are consistent with those reported in other studies [18,19,20]. Most of the existing literature focuses on adult care, as there is limited research on pediatric settings.

Continuing with the identified challenges, the nurses, like those in Madeleine Powell et al.’s study [17], emphasized that the timing of handovers is another significant hurdle. Effective coordination between healthcare professionals is essential for a smooth transfer of patient care. After a verbal handover, coupled with an inappropriate and non-standardized electronic record, nurses on the wards are left to “fend for themselves”. They must rely on medical discharge reports that omit critical nursing care details or ask parents for information, an approach that they view is unprofessional. To improve communication, the development of comprehensive nursing discharge reports is necessary. These reports should be tailored to clinical practice, and nurses should have input into their design to ensure they are user-friendly [40].

In addition to addressing the timing and content of handovers, the introduction of nursing discharge reports would resolve another key issue identified by the nurses: uncertainty about what information should be communicated. This problem has been noted in other studies, such as that by James S. et al. [15], which found that nurses often lacked clarity about the critical details to convey during handover.

We concur with other studies [24] that management teams must investigate why nurses perceive pediatric handovers to be inadequate. They should consider not only quantitative metrics but also the qualitative experience of nurses, as the perception that they are required to complete tasks merely for administrative purposes undermines their motivation. Providing healthcare professionals with optimal tools is essential to support and sustain their motivation [41].

To address these challenges, the nurses emphasized the importance of training. They all agreed on the need for practical, continuous education focused on patient transfer planning and the use of standardized, shared records, as recommended by other studies [16,18,42]. They also called for management to commit to ensuring that nurses receive this training, potentially through mandatory programs linked to incentives or financial rewards. Solving the problem of inadequate handovers is crucial for the nursing profession, as highlighted by another research [15,41]. A reliable record-keeping system that facilitates accurate and thorough documentation is essential, with both nurses and management taking responsibility for resolving this issue.

It is noteworthy that the role of the child-parent dynamic was not raised by the interviewed nurses, despite the potential anxiety parents may experience when transitioning to a new service with reduced care intensity. Further exploration of how parents perceive these moments would be valuable.

### 4.1. Future Research Directions

Building on the findings of this study, several future research avenues are suggested:

- Investigating the perceptions of middle and senior management regarding the handover process.
- Developing and testing a protocol for patient transfer and evaluating its implementation.
- Assessing the perceptions of patients and/or their parents following transfer.
- Exploring the views of nursing students on the use of standardized nursing methodologies.
- Conducting multicenter studies to compare with the strengths and weaknesses of other hospitals and develop a more effective implementation strategy.

### 4.2. Study Limitations

This study has several limitations:

- The topic is not widely discussed, making it challenging to conduct interviews.
- Given the potential for controversy with senior management, some interviews may not have fully captured the information sought. After 21 interviews, no new information was obtained, but the lack of researcher experience may have contributed to the omission of certain relevant topics.
- Focus groups could not be conducted, as nurses in pediatric units are familiar with one another, potentially influencing responses. Despite the use of individual interviews, interviewees still discussed their experiences with colleagues, which may have shaped their answers.
- Comparisons were primarily made with studies in adult settings, highlighting the need for more research focused specifically on pediatric care.

## 5. Conclusions

The nurses rated the handover process between different levels of care as inadequate and expressed a positive attitude toward the implementation of protocols to address existing communication issues. Currently, discharge practices vary by individual nursing staff, with no standardized system consistently known or applied by all professionals. This lack of a unified approach creates a communication gap among nursing professionals, underscoring the need for a formal discharge report that documents the care provided by nurses. Such a report would complement verbal handovers, promoting safer and higher-quality care for pediatric patients. Additionally, it is essential to establish a consensus on the timing of handovers to ensure they are conducted efficiently, with active involvement from the responsible nurses.

Nurses across departments have proposed regular meetings to define the essential information that should be conveyed during handover. This would facilitate the development of a standardized handover system tailored to the needs of different services. Given that handover is a critical point for patient safety, it is imperative that hospital management implements policies to support and facilitate its effective execution.

Management should urgently consider establishing committees dedicated to designing and implementing standardized transfer protocols, thereby promoting a culture of safety. Ensuring high-quality, systematic transfers is essential for safeguarding patient well-being. The implementation of standardized transfer systems is particularly important due to its benefits for patient safety, minimizing communication errors, and enhancing the quality of care.

## Figures and Tables

**Table 1:** Interview script.

Area to Investigate	Question Asked
Knowledge	Are you aware of different standardized transfer strategies and have you received information on this topic?
Current situation	How does the transfer take place in your hospital?
Use	What barriers or difficulties do you see? How could their use be improved? What do you think about the software available?
Usefulness in health care practice	Is it useful in healthcare practice, in what ways can it facilitate practice, and do you see benefits in its implementation?
Attitude and motivation of professionals	What is the attitude of the professionals, and do you think there would be better communication if you knew the staff of another service?
Forms of involvement	How could the involvement of professionals be encouraged?

## Data Availability

The data presented in this study are available upon request from the corresponding author due to privacy of the study participants. **Public Involvement Statement:** The nursing team participated on a voluntary basis after being informed of the purpose of the research through interviews. **Guidelines and Standards Statement:** This manuscript was drafted against the COREQ for qualitative research [32]. **Use of Artificial Intelligence:** AI or AI-assisted tools were not used in drafting any aspect of this manuscript.
